# Evaluation of an eight marker-panel including long mononucleotide repeat markers to detect microsatellite instability in colorectal, gastric, and endometrial cancers

**DOI:** 10.1186/s12885-023-11607-6

**Published:** 2023-11-13

**Authors:** Yousun Chung, Soo Kyung Nam, Ho Eun Chang, Cheol Lee, Gyeong Hoon Kang, Hye Seung Lee, Kyoung Un Park

**Affiliations:** 1 Department of Laboratory Medicine, Kangdong Sacred Heart Hospital, Seoul, Republic of Korea; 2 Department of Interdisciplinary Program in Cancer Biology, Seoul National University College of Medicine, Seoul, Republic of Korea; 3 Department of Laboratory Medicine, Seoul National University College of Medicine, Seoul, Republic of Korea; 4 Department of Pathology, Seoul National University College of Medicine, Seoul, Republic of Korea; 5 Department of Pathology, Seoul National University Hospital, 101 Daehak-ro, Jongno-gu, Seoul, 03080 Republic of Korea; 6 Department of Laboratory Medicine, Seoul National University Bundang Hospital, 82 Gumi-ro 173, Bundang-gu, Seongnam, 13620 Republic of Korea

**Keywords:** Microsatellite instability, Long mononucleotide repeat marker, Colorectal cancer, Gastric cancer, Endometrial cancer

## Abstract

**Background:**

Accurate determination of microsatellite instability (MSI) status is critical for optimal treatment in cancer patients. Conventional MSI markers can sometimes display subtle shifts that are difficult to interpret, especially in non-colorectal cases. We evaluated an experimental eight marker-panel including long mononucleotide repeat (LMR) markers for detection of MSI.

**Methods:**

The eight marker-panel was comprised of five conventional markers (BAT-25, BAT-26, NR-21, NR-24, and NR-27) and three LMR markers (BAT-52, BAT-59 and BAT-62). MSI testing was performed against 300 specimens of colorectal, gastric, and endometrial cancers through PCR followed by capillary electrophoresis length analysis.

**Results:**

The MSI testing with eight marker-panel showed 99.3% (295/297) concordance with IHC analysis excluding 3 MMR-focal deficient cases. The sensitivity of BAT-59 and BAT-62 was higher than or comparable to that of conventional markers in gastric and endometrial cancer. The mean shift size was larger in LMR markers compared to conventional markers for gastric and endometrial cancers.

**Conclusions:**

The MSI testing with eight maker-panel showed comparable performance with IHC analysis. The LMR markers, especially BAT-59 and BAT-62, showed high sensitivity and large shifts which can contribute to increased confidence in MSI classification, especially in gastric and endometrial cancers. Further study is needed with large number of samples for the validation of these LMR markers.

**Supplementary Information:**

The online version contains supplementary material available at 10.1186/s12885-023-11607-6.

## Background

 Microsatellites are difficult to be replicated with high fidelity because of slippage of the polymerase on repeating units. This results in a change in allele length due to either insertion or deletion of the units [1]. These replication errors are immediately recognized and repaired by mismatch repair (MMR) proteins. However, when there is MMR deficiency (dMMR) in tumors, these errors cannot be corrected, resulting in microsatellite instability (MSI) [2–4]. dMMR arises from the inactivation of MMR genes by hypermethylation and epigenetic silencing of MLH1 in most sporadic tumors [5] or by germline mutation in one of the MMR genes in most Lynch syndrome cases [6]. dMMR occurs in approximately 15–20% of colorectal cancers, in addition to many other cancer types, such as gastric, endometrial, and pancreatic cancer [6–10].

dMMR assays are used to screen patients with solid tumors for Lynch syndrome and immune checkpoint inhibitor therapy [11–13]. Pembrolizumab (Merck, Kenilworth, NJ), a PD-1 inhibitor, was approved by the US Food and Drug Administration (FDA) in 2017 for the cases of unresectable or metastatic solid tumors with MSI-high (MSI-H) by PCR-based MSI assays or loss of MMR expression by immunohistochemical (IHC) staining, independent of PD-L1 expression, tissue type, and tumor site [14]. Accurate determination of the dMMR status is critical for the optimal treatment of patients with various cancers.

The two major categories of tissue-based testing currently in use for the detection of the status of MMR are immunohistochemistry (IHC) and PCR-based assays. IHC analysis is used to evaluate the loss of expression for major MMR proteins (MLH1, MSH2, MSH6, and PMS2) to determine whether a tumor shows dMMR. PCR-based MSI testing detects changes in allelic lengths in a panel of microsatellite markers through PCR, followed by capillary electrophoresis length analysis. The Bethesda panel has been conventionally used for MSI detection: two mononucleotide repeat markers (BAT-25 and BAT-26) and three dinucleotide repeat markers (D2S123, D5S346, and D17S250) [11]. The number in the mononucleotide marker name indicates the number of poly-A repeats based on the GenBank GRCh38 reference genome assembly. Recently, a pentaplex panel with five mononucleotide repeat markers (BAT-25, BAT-26, NR-21, NR-24, and NR-27) was developed and is considered the current standard for PCR-based MSI assays [15–20].

Conventional markers can sometimes display subtle shifts that are difficult to interpret, especially in non-colorectal cases [21–23]. Long mononucleotide repeat (LMR) markers, which are typically 52 to 60 adenine repeats, are more prone to replication errors and, therefore, may improve the sensitivity of MSI assays [24–26]. Several studies have evaluated the performance of LMR markers in MSI testing. In a study with LMR markers of BAT-52, BAT-55, BAT-56, BAT-57, and BAT-59 [19] and another study with BAT-52, BAT-56, BAT-59, and BAT-60, [27] an increased detection of MSI-H lesions was observed over that of conventional markers in colorectal cancer. Another recent study with BAT-52, BAT-56, BAT-59, and BAT-60 showed greater sensitivity of LMR markers for detecting dMMR in endometrial cancer [28].

In this study, we aimed to evaluate the performance of a laboratory-developed eight-marker panel consisting of five widely used conventional markers (BAT-25, BAT-26, NR-21, NR-24, and NR-27) and three LMR markers (BAT-52, BAT-59, and BAT-62) for MSI detection in colorectal, gastric, and endometrial cancers.

## Methods

### Samples

The Seoul National University Hospital Institutional Review Board approved this study (H-2112-168-1286), and all methods were performed in accordance with the relevant guidelines and regulations. One hundred patients with colorectal, gastric, and endometrial cancers who underwent surgical resection at Seoul National University Hospital were included in this study. The selection criteria included patients with results of MMR protein expression by IHC and MSI status by PCR-based MSI analysis with National Cancer Institute (NCI) panel at the time of diagnosis for colorectal and gastric cancers. For patients with endometrial cancer, PCR-based MSI analysis with NCI panel was not routinely performed, and patients with MMR protein expression results by IHC were included. Informed consent was obtained from all subjects or their legal guardians. Archived formalin-fixed paraffin-embedded (FFPE) samples were collected.

### Immunohistochemistry analysis

IHC analysis was performed using anti-MLH1 (mouse monoclonal primary antibody, prediluted, clone M1; Ventana, Tucson, AZ, USA), anti-MSH2 (mouse monoclonal primary antibody, prediluted, clone G219-1129; Ventana, USA), anti-MSH6 (mouse monoclonal primary antibody, 1:50, clone 44; Cell Marque, Rocklin, CA, USA), and anti-PMS2 (mouse monoclonal primary antibody, prediluted, clone ERP3947; Ventana, USA). Samples were graded for the absence or presence of nuclear staining of MMR proteins in tumor cells compared to internal positive controls, such as non-neoplastic epithelial cells, stromal cells, and lymphocytes, in the vicinity of the tumor. Each case was classified into one of three categories according to the intensity and proportion of tumor nuclear staining in the whole slide as follows: [1] “intact expression” was defined as unequivocal nuclear staining in all tumor cells with clear staining of internal control tissue adjacent to the tumor cells, [2] “loss of expression” was defined as unequivocal loss of nuclear staining in all tumor cells with clear staining of internal control tissue in the vicinity of the tumor, and [3] “focal loss of expression” was defined as clearly demarcated regional loss of tumor nuclear staining with internal control tissue adjacent to the tumor cells showing clear staining.

### Microsatellite instability analysis using National Cancer Institute (NCI) panel

DNA was extracted from macro-dissected FFPE tumor tissue slides and from matching normal FFPE tissue using the Maxwell 16 FFPE plus LEV DNA purification kit (Promega, Madison, WI, USA). MSI analysis was performed using NCI panel of five markers, including two mononucleotide markers (BAT-26 and BAT-25) and three dinucleotide markers (D5S346, D17S250, and D2S123). PCR was performed with 10µL of each reaction mixture in a set of mononucleotides and dinucleotides using a SimpliAmp thermal cycler (Applied Biosystems, Foster City, CA, USA) according to the following protocol: 5 min at 95 °C for polymerase activation; 34 cycles at 95 °C for 30 s, 52 °C for 30 s, 65 °C for 60 s, followed by an additional 10 min at 72 °C. The PCR products were analyzed using an automated sequencer (ABI 3730xl DNA Analyzer; Applied Biosystems, Foster City, CA, USA) and automated sizing of the alleles found using Data Collection v3.0 software (Applied Biosystems). Allelic sizes for matching normal and tumor samples were compared, and MSI was considered unstable if there was a shift of 2 bp or more in the tumor allele. Samples were classified as MSI-H when two or more markers were unstable, MSI-L when one marker was unstable, or MSS when there were no unstable markers.

### Microsatellite instability analysis using pentaplex panel (BAT-25, BAT-26, NR-21, NR-24, and NR-27)

DNA was extracted from macro-dissected FFPE tumor tissue slides and matching normal FFPE tissue using the Maxwell RSC FFPE Plus DNA Kit (Promega, Madison, WI, USA). The DNA concentration was quantified using a Nanodrop (Thermo Scientific, Wilmington, DE, USA). MSI analysis was performed with 5 conventional mononucleotide repeat markers: NR-21, NR-24, NR-27, BAT-25, and BAT-26. PCR was performed using a SimpliAmp thermal cycler (Applied Biosystem, Foster City, CA) in 25 µL reaction mixtures containing 30 ng of extracted tissue DNA, 10 pmol/µL of each set of primers labelled with fluorescence, and a 2X Platinum II Hot Start PCR Master mix (Invitrogen) according to the following protocol: 3 min at 95 °C for polymerase activation; 40 cycles at 94 °C for 15 s, 58 °C for 15 s, and 72 °C for 30 s; followed by an additional 5 min at 72 °C. The PCR product was evaluated using a SeqStudio Genetic Analyzer (Applied Biosystems, CA, USA), based on automated capillary electrophoresis and automated sizing of the alleles found using GeneMapper Software 6 (microsatellite analysis mode, Applied Biosystems, USA). Allelic sizes for matching normal and tumor samples were compared, and MSI was considered unstable if there was a shift of 2 bp or more in the tumor allele. Samples were classified as MSI-H when two or more markers were unstable, MSI-L when one marker was unstable, or MSS when there were no unstable markers.

### Microsatellite instability analysis using experimental panel including LMR markers (NR-21, NR-24, NR-27, BAT-25, BAT-26, BAT-52, BAT-59, and BAT-62)

DNA was extracted from macro-dissected FFPE tumor tissue slides and matching normal FFPE tissue using the Maxwell RSC FFPE Plus DNA Kit (Promega). The DNA concentration was quantified using a Nanodrop (Thermo Scientific). MSI analysis was performed using five conventional mononucleotide repeat markers and three additional long mononucleotide repeat markers: NR-21, NR-24, NR-27, BAT-25, BAT-26, BAT-52, BAT-59, and BAT-62. DNA extraction and PCR were performed using the same protocol used for the conventional five-marker panel. The PCR product was evaluated using a SeqStudio Genetic Analyzer (Applied Biosystems, CA, USA), based on automated capillary electrophoresis and automated sizing of the alleles found using GeneMapper Software 6 (microsatellite analysis mode, Applied Biosystems, USA). Allelic sizes for matching normal and tumor samples were compared, and MSI was considered unstable if there was a shift of 2 bp or more in the tumor allele. Samples were classified as MSI-H when three or more (≥ 30%) markers out of a panel of eight were unstable, MSI-L when one or two of the markers were unstable, and MSS when there were no unstable markers.

### Statistical analysis

Statistical analyses were performed using McNemar’s test to compare differences in sensitivity and specificity between the markers for each cancer type. For the size of allele shift, Tukey’s method with one-way ANOVA was used to compare markers for each cancer type, and t-test with one-way ANOVA was used to compare cancer types for each marker. The size of allelic changes between the patterns of deficient mismatch repair proteins was compared using the Kruskal–Wallis test. All statistical analyses were performed using R 4.2.1, and *P* values less than 0.05 were considered significant.

## Results

### Comparison of the results between IHC analysis and PCR-based MSI analysis

The results of IHC analysis and PCR-based MSI analysis are compared in Table 1 for colorectal, gastric, and endometrial cancers.

**Table 1:** Comparison of the results by IHC analysis and those by PCR-based MSI analysis for colorectal, gastric, and endometrial cancers

Cancer types	IHC analysis	NCI panel			Pentaplex panel			Experimental panel including LMR markers		
		MSS	MSI-L	MSI-H	MSS	MSI-L	MSI-H	MSS	MSI-L	MSI-H
Colorectal cancer	dMMR	0	0	36	0	0	36	0	0	36
	fMMR	0	0	1	0	0	1	0	0	1
	pMMR	30	30	3^a^	62	1	0	57	6	0
Gastric cancer	dMMR^b^	0	0	28	0	0	29	0	0	29
	fMMR	0	1	0	0	0	1	0	0	1
	pMMR^b^	63	6	0	66	4	0	57	12	1^c^
Endometrial cancer	dMMR	NT	NT	NT	1	1^d^	23	0	1^e^	24
	fMMR	NT	NT	NT	0	0	1	0	0	1
	pMMR	NT	NT	NT	73	1	0	66	8	0

*Abbreviations*: *IHC* Immunohistochemical, *LMR* Long mononucleotide repeat, *MSI* Microsatellite instability, *NT* Not tested

^a^Two dinucleotide markers (D17S250 and D2S123) were unstable

^b^PCR-based MSI analysis using NCI panel was not performed for one dMMR case and one pMMR case of gastric cancer

^c^Three markers (BAT-25, BAT-59, and BAT-62) showed instability

^d^Only NR-21 showed instability

^e^Only BAT-59 showed instability

1) IHC analysis vs. MSI analysis using the NCI panel (BAT-25, BAT-26, D2S123, D5S346, and D17S250).

As MSI analysis was routinely performed at the time of diagnosis for colorectal and gastric cancers using the NCI panel, the results were compared with those from IHC analysis.

Among the total of 100 cases of colorectal cancer, 36 cases were assessed as dMMR, 1 case as MMR-focal deficient (fMMR), and 63 cases as MMR-proficient (pMMR) by IHC analysis. MSI analysis using the NCI panel classified all 36 dMMR cases as MSI-H. The fMMR case was classified as MSI-H by MSI analysis, as all five markers showed instability. Among the 63 pMMR cases, 30 were classified as MSS and another 30 as MSI-L. Three pMMR cases were classified as MSI-H, as two dinucleotide markers (D17S250 and D2S123) showed instability. The concordance rate between IHC and MSI results using the NCI panel, in which pMMR cases were classified as MSS or MSI-L, and dMMR cases as MSI-H, was 97.0% (96/99) for colorectal cancer, excluding the fMMR case.

Among the 100 cases of gastric cancer, 29 cases were assessed as dMMR, 1 case as fMMR, and 70 cases as pMMR by IHC analysis. MSI analysis using the NCI panel was not performed for 1 dMMR and 1 pMMR case. The remaining 28 dMMR cases were classified as MSI-H by MSI analysis using the NCI panel. The fMMR case was classified as MSI-L by MSI analysis, as only BAT-25 exhibited instability. Among the 69 pMMR cases, 63 were classified as MSS and the other 6 cases as MSI-L. The concordance rate between the IHC and MSI results using the NCI panel was 100% (97/97) for gastric cancer, excluding the fMMR case.

2) IHC analysis vs. MSI analysis using the pentaplex panel (BAT-25, BAT-26, NR-21, NR-24, and NR-27).

For colorectal cancer, MSI analysis using the pentaplex panel classified all 36 dMMR cases as MSI-H. The fMMR case was classified as MSI-H by MSI analysis, as all five markers showed instability. Among the 63 pMMR cases, 62 were classified as MSS and one other case as MSI-L. The concordance rate between the IHC and MSI results using the pentaplex panel was 100% (99/99) for colorectal cancer, excluding the fMMR case.

For gastric cancer, MSI analysis using the pentaplex panel classified all 29 dMMR cases as MSI-H. The fMMR case was classified as MSI-H by MSI analysis, as BAT-25, BAT-26, and NR-24 exhibited instability. Among the 70 pMMR cases, 66 were classified as MSS and the four as MSI-L. For gastric cancer, the concordance rate between the IHC and MSI results using the pentaplex panel was 100% (99/99), excluding the fMMR case.

Lastly, among the 25 dMMR endometrial cancer cases, MSI analysis using the pentaplex panel classified 23 cases as MSI-H, except one case in which none of the five markers showed instability and the other case in which only NR-21 showed instability. The fMMR case was classified as MSI-H based on MSI analysis, with all markers showing instability. Among the 74 pMMR cases, 73 were classified as MSS and one other case as MSI-L. The concordance rate between IHC and MSI results using the pentaplex panel was 98.0% (97/99) for endometrial cancer, excluding the fMMR case.

3) IHC analysis vs. MSI analysis using LMR markers (BAT-25, BAT-26, NR-21, NR-24, NR-27, BAT-52, BAT-59, and BAT-62).

For colorectal cancer, MSI analysis by the experimental panel classified all 36 MMR-deficient cases as MSI-H. The fMMR case was classified as MSI-H by MSI analysis as all eight markers showed instability. Among the 63 pMMR cases, 57 were classified as MSS and the other 6 cases as MSI-L. The concordance rate between IHC and MSI results using the experimental panel was 100% (99/99) for colorectal cancer, excluding the fMMR case.

For gastric cancer, MSI analysis by the experimental panel classified all 29 dMMR cases as MSI-H. The fMMR case was classified as MSI-H by MSI analysis, as BAT-25, BAT-26, NR-24, and all three LMR markers exhibited instability. Among the 70 pMMR cases, 57 were classified as MSS, and 12 were classified as MSI-L. One pMMR case was classified as MSI-H, as BAT-26, BAT-59, and BAT-62 showed instability. The concordance rate between the IHC and MSI results using the experimental panel was 99.0% (98/99) for gastric cancer, excluding the fMMR case.

Lastly, for endometrial cancer, among the 25 dMMR cases, MSI analysis by experimental panel classified 24 cases as MSI-H, except for one case in which only BAT-59 out of eight markers showed instability. The fMMR case was classified as MSI-H based on MSI analysis, with all markers showing instability. Among the 74 pMMR cases, 66 were classified as MSS and the other 8 cases as MSI-L. For endometrial cancer, the concordance rate between IHC and MSI results using the experimental panel was 99.0% (98/99), excluding the fMMR case.

### Sensitivity and specificity of individual markers

The sensitivity and specificity for the detection of MSI for each marker were evaluated based on the results of IHC analysis and MSI analysis with pentaplex panel (Table 2). The sensitivity of each marker was calculated as the percentage of cases in which the marker showed instability among the cases classified as dMMR by IHC analysis or MSI-H by MSI analysis. Specificity was calculated as the percentage of cases in which the marker showed stability among the cases classified as pMMR by IHC analysis and MSS/MSI-L by MSI analysis. For colorectal cancer cases, BAT-25, BAT-26, and BAT-59 showed instability in all dMMR/MSI-H cases, whereas each of the other markers missed only one dMMR case. For gastric cancer, BAT-59 (100%) showed the highest sensitivity followed by BAT-25 (96.7%), BAT-26 (96.7%), BAT-62 (96.7%), NR-21 (93.3%), NR-24 (93.3%), BAT-52 (93.1%), and NR-27 (90.0%). For endometrial cancer, BAT-59 (96.2%) showed the highest sensitivity followed by NR-27 (92.3%), BAT-25 (84.6%), BAT-26 (84.6%), BAT-62 (84.6%), NR-21 (80.8%), NR-24 (76.9%), and BAT-52 (65.4%). There was no statistically significant difference in sensitivity between the markers for colorectal and gastric cancers. For endometrial cancer, only BAT-52 showed statistically significant lower sensitivity than BAT-59 (*P* = 0.013) and NR-27 (*P* = 0.046). In terms of specificity, most markers showed high specificity (> 95%), except BAT-52 (93.2%) in endometrial cancer and BAT-59 (88.6%) in gastric cancer. There was no statistically significant difference in specificity between the markers for colorectal and endometrial cancer. For gastric cancer, only BAT-59 exhibited a lower specificity than NR-21 and NR-24, both of which had a specificity of 100%, and BAT-25 (*P* = 0.046) and NR-27 (*P* = 0.046), with statistical significance.

**Table 2:** Sensitivity and specificity of the individual markers in the experimental panel

Marker	Colorectal cancer		Gastric cancer		Endometrial cancer	
	Sensitivity	Specificity	Sensitivity	Specificity	Sensitivity	Specificity
BAT-25	100	100	96.7	98.6	84.6	100
BAT-26	100	100	96.7	97.1	84.6	100
NR-21	97.3	100	93.3	100	80.8	98.6
NR-24	97.3	100	93.3	100	76.9	100
NR-27	97.3	98.4	90.0	98.6	92.3	100
BAT-52	97.3	95.2	93.3	97.1	65.4	93.2
BAT-59	100	98.4	100	88.6	96.2	97.3
BAT-62	97.3	98.4	96.7	95.7	84.6	98.6

### Comparison between markers of the size of allelic changes

**Fig. 1:**
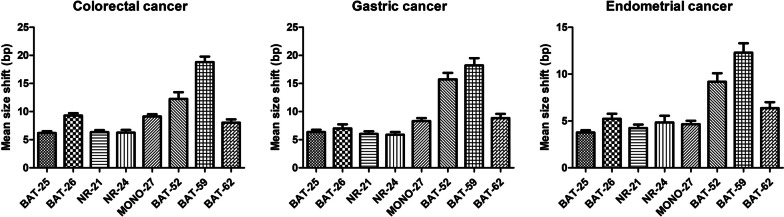
The average allele size changes for the markers in the experimental panel in 100 cases each of colorectal, gastric, and endometrial cancer. Error bars show the standard error of the mean. The mean size of allelic changes was greater with BAT-52 and BAT-59 than with the conventional markers

Allelic sizes for tumor samples were compared to those for matching normal samples (Fig. 1), and the shift in allele sizes was evaluated in base pairs (Fig. 1).

The mean size of allelic changes was greater with BAT-52 and BAT-59 than with the conventional markers (BAT-25, BAT-26, NR-21, NR-24, and NR-27) (Table 3). BAT-59 showed a greater shift than conventional markers, with statistical significance for all cancer types. For BAT-52, there were statistically significant differences in all the conventional markers in gastric and endometrial cancer, whereas there were significant differences only with BAT-25, NR-21, and NR-24 in colorectal cancer. In the case of BAT-62, the mean size of allelic changes was greater than that of conventional markers in gastric and endometrial cancers, although the differences were not statistically significant. When the shift size was compared between cancer types, the mean shift size in endometrial cancer was less than that in colorectal and gastric cancers for all the markers (Table 3).

**Table 3:** The size of allelic changes for MSI detection in the individual markers of the experimental panel for colorectal, gastric, and endometrial cancers

Marker	Mean (standard deviation)						*P*-value		
	Colorectal cancer		Gastric cancer		Endometrial cancer		Colorectal vs. gastric cancers	Endometrial vs. Colorectal cancers	Endometrial vs. gastric cancers
BAT-25	6.0	(1.8)	6.3	(2.3)	3.5	(1.3)	0.573	<0.001^*^	<0.001^*^
BAT-26	9.2	(2.7)	6.5	(4.1)	5.1	(2.6)	0.003^*^	<0.001^*^	0.083
NR-21	6.4	(2.2)	6.0	(2.4)	3.8	(2.0)	0.518	<0.001^*^	0.001^*^
NR-24	6.0	(2.8)	5.7	(2.5)	4.5	(3.1)	0.677	0.064	0.127
NR-27	9.1	(2.3)	8.1	(2.7)	4.4	(1.9)	0.165	<0.001^*^	<0.001^*^
BAT-52	11.4	(6.9)	15.7	(6.3)	9.0	(4.1)	0.009^*^	0.094	<0.001^*^
BAT-59	18.6	(5.9)	17.9	(7.9)	12.3	(5.1)	0.661	<0.001^*^	<0.001^*^
BAT-62	7.7	(3.3)	9.1	(4.4)	6.0	(2.8)	0.171	0.058	0.005^*^

^*^The *P* values were less than 0.05, using the t-test with one-way ANOVA

As LMR markers can exhibit heterozygosity (Supplemental Figure S1), unlike conventional markers which are quasimonomorphic mononucleotide repeats, we investigated the distribution of marker size in each LMR marker using 300 cases of normal tissues (Supplemental Figure S2). The difference in the number of repeats between alleles in the cases of heterozygosity was found to be 5 bps or more, substantially decreasing the chances of overlooking MSI cases resulting from loss of heterozygosity (LOH). The heterozygosity levels for each LMR marker were as follows: 24.3% for BAT-52, 58.3% for BAT-59, and 42.3% for BAT-62.

### The size of allelic changes according to IHC patterns

**Fig. 2:**
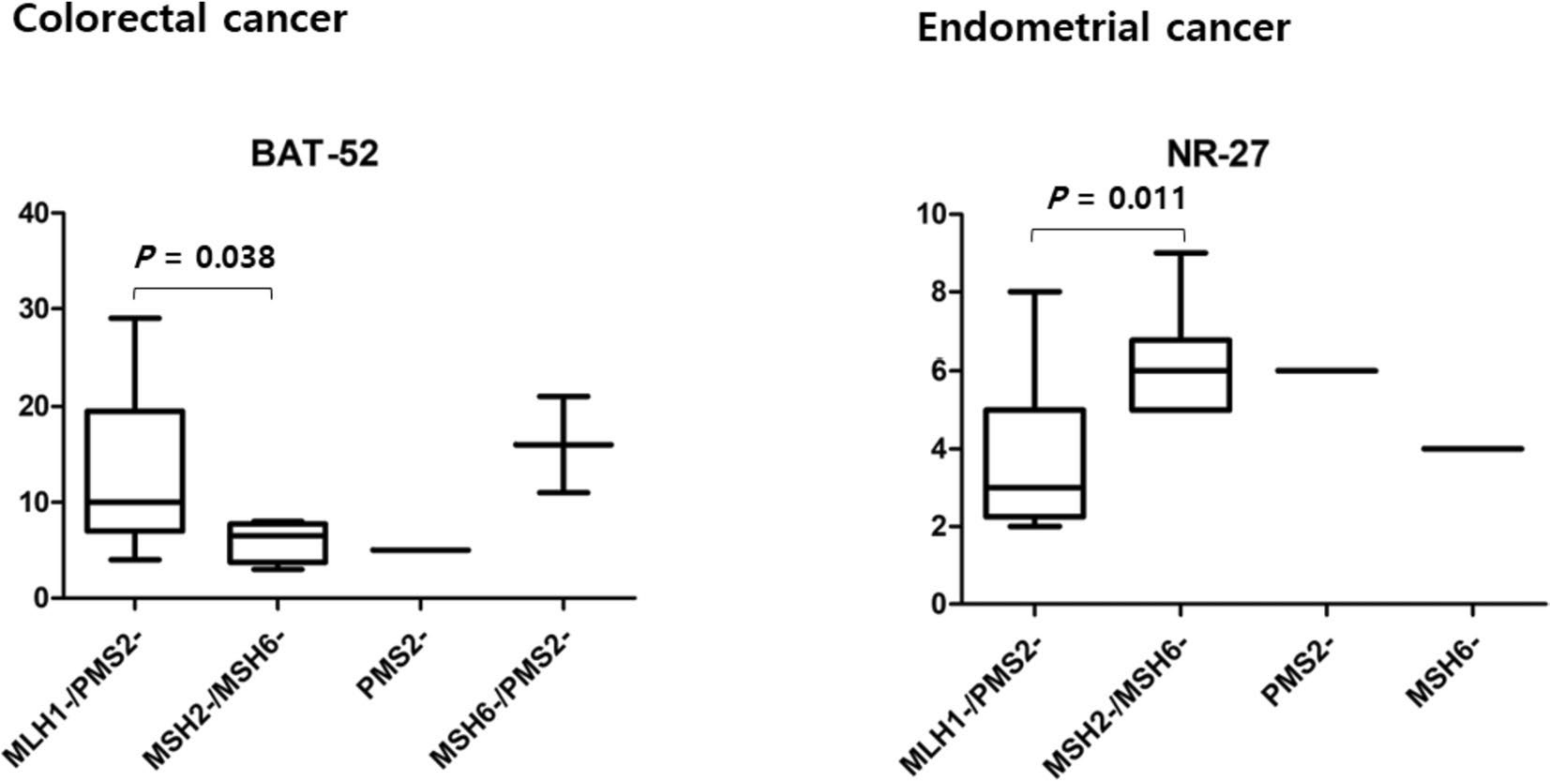
Box and whiskers plot showing the median, max, min and 1st and 3rd quartile of the size of allelic changes for MSI detection in individual marker according to the patterns of deficient mismatch repair proteins. There was a statistically significant difference between MLH1-/PMS2- and MSH2-/MSH6- for BAT-52 in colorectal cancer (*P* = 0.038), and for NR-27 in endometrial cancer (*P* = 0.011), using the Kruskal–Wallis test

IHC analysis demonstrated that MLH1-/PMS2- was the most prevalent pattern in all cancer types (Table 4). In colorectal cancer, the MLH1-/PMS2- pattern was observed in 81.1% of the cases, followed by MSH2-/MSH6- (10.8%), MSH6-/PMS2- (5.4%), and PMS2- (2.7%). In gastric cancer, the MLH1-/PMS2- pattern was observed in 93.3% of cases, followed by PMS2- (3.3%), and MLH1-/MSH6-/PMS2- (3.3%). In endometrial cancer, the MLH1-/PMS2- pattern was observed in 69.2% of the cases, followed by MSH2-/MSH6- (23.1%), PMS2- (3.8%), and MSH6- (3.8%). The difference in the distribution of these dMMR patterns among the different cancer types was statistically significant (*P* = 0.026). When the size of allelic changes was compared between IHC patterns in each cancer type (Supplemental Figure S3), there was a statistically significant difference between MLH1-/PMS2- and MSH2-/MSH6- for BAT-52 in colorectal cancer (*P* = 0.038) and for NR-27 in endometrial cancer (*P* = 0.011) (Fig. 2).

**Table 4:** Distribution (%) of IHC patterns for the dMMR cases of colorectal, gastric, and endometrial cancers

IHC pattern	Colorectal cancer		Gastric cancer		Endometrial cancer	
MLH1-/PMS2-	81.1	(30/37)	93.3	(28/30)	69.2	(18/26)
MSH2-/MSH6-	10.8	(4/37)	0.0	(0/30)	23.1	(6/26)
PMS2-^a^	2.7	(1/37)	3.3	(1/30)	3.8	(1/26)
MSH6-/PMS2-	5.4	(2/37)	0	(0/30)	0	(0/26)
MLH1-/MSH6-/PMS2-	0	(0/37)	3.3	(1/30)	0	(0/26)
MSH6-^a^	0	(0/37)	0.0	(0/30)	3.8	(1/26)

^a^All other MMR proteins were intact

## Discussion

This study evaluated the performance of an eight- marker panel consisting of five conventional markers (BAT-25, BAT-26, NR-21, NR-24, and NR-27) and three LMR markers (BAT-52, BAT-59, and BAT-62) for MSI detection in colorectal, gastric, and endometrial cancers. Compared to the results of IHC analysis for MMR protein expression, the concordance between IHC and MSI results using the experimental panel was 99.3% (295/297), excluding fMMR cases. The discordant case was one pMMR gastric cancer which was classified as MSI-H by MSI testing with BAT-26, BAT-59, and BAT-62 showing instability. This discrepancy could result from the false negativity of IHC staining and the improved dMMR detection of LMR markers. False-negative IHC results can be caused by retained epitopes in non-functional proteins that may still be antigenically detectable or by various technical and interpretive problems [29–32]. The other discrepant case was a dMMR endometrial case which was classified as MSI-L by MSI testing with only BAT-59 of the eight showing instability. Previous reports have suggested that neoadjuvant therapy can induce the loss of MMR protein expression [33, 34]. However, since the IHC analysis was performed at the time of diagnosis, the likelihood of false negativity in the IHC analysis due to this cause is considered to be very low in this study. It is well known that conventional markers show lower sensitivity for dMMR detection in endometrial cancer than in colorectal cancer [21, 22, 35, 36]. Although the experimental panel failed to classify this dMMR case as MSI-H, this result highlights the usefulness of LMR markers, especially BAT-59, for MSI testing in endometrial cancer

 We also compared the results of MSI classification by the experimental panel to those of other PCR-based MSI analyses, the NCI panel, and the pentaplex panel. In colorectal cancer, three pMMR cases were classified as MSI-H by the NCI panel, with two dinucleotide markers showing instability, whereas two other panels classified them as MSS. Tumors in which only dinucleotides are unstable are often misclassified as MSI-H by the NCI panel and typically show MMR expression by IHC, as in these three cases [11, 37]. For the fMMR case in gastric cancer, the NCI classified it as MSI-L, with only BAT-25 showing instability, whereas the other two panels classified it as MSI-H. There was a discrepancy in the result of BAT-26, and a possible explanation for this is intratumor heterogeneity which is a distinct genetic alteration between tumor cells [38]. Between the pentaplex panel and the experimental panel with LMR markers, there were two discrepant cases: the above-mentioned pMMR gastric case and the dMMR endometrial case. For the pMMR gastric case, the experimental panel classified it as MSI-H, as BAT-59 and BAT-62 showed instability in addition to BAT-26, whereas the pentaplex panel classified it as MSI-L. For the dMMR endometrial case, the experimental panel classified it as MSI-H, BAT-52 and BAT-59 showed instability in addition to NR-21, whereas the pentaplex panel classified it as MSI-L. We could not determine the exact MSI status in these discrepant cases using other approaches. Further studies with a larger cohort with known MSI status are needed to determine whether LMR markers have superior sensitivity to conventional markers

At the individual marker level, the high-sensitivity rankings varied by cancer type. For colorectal cancer, all markers showed high sensitivity, demonstrating that the MSI testing method was developed and designed primarily for colorectal cancer from the beginning. For gastric and endometrial cancers, BAT-59 showed the highest relative sensitivity, highlighting the usefulness of this LMR marker for the detection of MSI in non-colorectal cancers. Previous reports have also demonstrated an increase in the detection of MSI-H lesions by LMR markers over conventional markers [19, 25, 26, 28]

In most clinical laboratories, the same marker panel for MSI testing is used, regardless of the tumor type. However, as the general marker panel had been developed and calibrated primarily for colorectal cancer and differences exist in the MSI profiles between tumor types, such a unified system may not be optimal for MSI detection. Many studies have reported lower sensitivity in detecting MSI in non-colorectal cancers using conventional panels [21, 22, 35, 36]. For efficient and optimal MSI testing for each cancer type, various panel configurations should be considered. Among the LMR markers investigated in this study, BAT-59 and BAT-62 can be utilized for MSI testing in gastric and endometrial cancers as they showed relatively high sensitivity

LMR markers generally result in larger allele size changes which can contribute to increased confidence in MSI classification by reducing the number of ambiguous calls associated with subtle shifts. Previous studies also showed that the size of allelic changes was significantly greater with LMR markers than with conventional ones [19, 28]. BAT-59 showed the largest change in allele size, followed by BAT-52 and BAT-62. Notably, the mean size shift in endometrial cancer was less than that in colorectal or gastric cancers for all markers with statistical significance except NR-24. With high sensitivity, it could be inferred that BAT-59 and BAT-62 should be included in the MSI testing panel, especially for non-colorectal cancers. Although BAT-52 showed a larger shift than conventional markers, its sensitivity was too low to be considered a candidate marker in MSI testing

We additionally analyzed the size of allelic changes in each marker according to IHC patterns to investigate whether there was any difference in MSI profiles according to IHC patterns. The distribution of IHC patterns in dMMR cases differed among colorectal, gastric, and endometrial cancers. Notably, the prevalence of the MLH1-/PMS2- pattern was lower in endometrial cancer (69.2%) than in colorectal (81.1%) and gastric cancers (93.3%). In a previous study which analyzed the shift size of conventional mononucleotide repeat markers according to IHC patterns in gastrointestinal cancers, [39] significant differences were observed in BAT-26 (between MLH1-/PMS2- and MSH6- and between MLH1-/PMS2- and PMS2-), NR-21 (between MLH1-/PMS2- and MSH6-), and NR-24 (between MLH1-/PMS2- and MSH6-), results of which are different from the findings of this study. Further studies with larger cohorts are needed to elucidate the association between MSI profiles and IHC patterns. Understanding the varying sizes of allelic changes among markers according to the IHC pattern can contribute to accurate determination of MSI status

Recently, NGS-based approaches are widely used to evaluate mutation of *MMR* genes, to evaluate MSI status using large panel of microsatellites, or to detect elevated tumor mutational burden (TMB) [40, 41]. Many studies found that high TMB is strongly correlated with MSI-H or dMMR [42–45]. In this study, TMB data was available in 17 cases, consisting of 3 dMMR and 5 pMMR cases of colorectal cancer, 1 pMMR case of gastric cancer, and 1 dMMR and 7 pMMR cases of endometrial cancer. All the four dMMR cases exhibited high TMB and MSI-H. Twelve pMMR cases showed low TMB and MSS/MSI-L, and the remaining one pMMR/MSS case of endometrial cancer which had a *POLE* mutation displayed high TMB. An elevated TMB in MSS/MSI-L cases can be due to other gene defects, such as *POLD1, POLE, MUTYH*, or other DNA repair genes [46, 47]. There was a statistically significant difference in the TMB results between the dMMR and pMMR groups, excluding the case with *POLE* mutation (Supplemental Figure S4).

A limitation of this study is that we could not determine the exact MSI status in discrepant cases using other approaches. Accordingly, we calculated the sensitivity of each marker based on the results of IHC analysis and MSI analysis using pentaplex panel which have been reported to be superior to NCI panels and are being widely used [15, 20]. Second, only size shifts of ≥ 2 bp were considered as showing instability which might have caused some unstable cases to be missed. However, very subtle size shifts of one base pair were observed in the pMMR cases and were considered possibly due to polymerase slippage. In addition, this subtle shift may result in inter-observer variability, reducing the reliability of MSI testing. To minimize this noise, we adopted only size shifts of ≥ 2 bp as criteria for the determination of instability in this study. Finally, the number of cases, especially dMMR cases, used in the evaluation was not sufficiently large for each cancer type. Further studies with a larger number of cases are warranted to validate LMR markers.

## Conclusions

We evaluated the performance of an eight-marker panel including LMR markers, in MSI detection. Determining tumor MSI status is critical for the diagnosis of Lynch syndrome and for determining treatment options for patients with various solid tumors. The LMR markers, especially BAT-59 and BAT-62, showed high sensitivity and large shifts which can contribute to increased confidence in the MSI classification. Further studies are necessary to validate these markers using a greater number of samples to elucidate their superiority over conventional markers.

### Supplementary Information

**Additional file 1: Supplemental Figure S1.** Representative electropherogram of a normal tissue showing heterozygosity in all long mononucleotide repeat markers. **Supplemental Figure S2.** The distribution of marker size in each long mononucleotide repeat (LMR) marker (BAT-52, BAT-59, and BAT-62) using 300 cases of normal tissues. **Supplemental Figure S3.** Box and whiskers plot showing the median, max, min and 1st and 3rd quartile of the size of allelic changes for MSI detection in individual marker according to the patterns of deficient mismatch repair proteins. There was a statistically significant difference between MLH1-/PMS2- and MSH2-/MSH6- for BAT-52 in colorectal cancer (*P* = 0.038) and for NR-27 in endometrial cancer (*P*= 0.011), using the Kruskal–Wallis test. **Supplemental Figure S4.** There was a statistically significant difference in the tumor mutational burden (TMB) results between dMMR and pMMR groups, excluding the case with POLE mutation (*P* = 0.003 by Mann-Whitney U test).

## Data Availability

The datasets used and/or analysed during the current study are available from the corresponding author on reasonable request.

## Abbreviations

MMR: Mismatch repair
dMMR: MMR deficiency
MSI: Microsatellite instability
FDA: US Food and Drug Administration
MSI-H: MSI-high
IHC: Immunohistochemical
LMR: Long mononucleotide repeat
FFPE: Formalin-fixed paraffin-embedded
fMMR: MMR-focal deficient
pMMR: MMR-proficient
