# Supplementary material for: Evaluation of an eight marker-panel including long mononucleotide repeat markers to detect microsatellite instability in colorectal, gastric, and endometrial cancers
Source: BMC Cancer. 2023 Nov 13;23:1100. doi: 10.1186/s12885-023-11607-6 (PMC10641958; doi:10.1186/s12885-023-11607-6)
Supplement: Supplementary file 1 — Additional file 1: Supplemental Figure S1. Representative electropherogram of a normal tissue showing heterozygosity in all long mononucleotide repeat markers. Supplemental Figure S2. The distribution of marker size in each long mononucleotide repeat (LMR) marker (BAT-52, BAT-59, and BAT-62) using 300 cases of normal tissues. Supplemental Figure S3. Box and whiskers plot showing the median, max, min and 1st and 3rd quartile of the size of allelic changes for MSI detection in individual marker according to the patterns of deficient mismatch repair proteins. There was a statistically significant difference between MLH1-/PMS2- and MSH2-/MSH6- for BAT-52 in colorectal cancer (P = 0.038) and for NR-27 in endometrial cancer (P= 0.011), using the Kruskal–Wallis test. Supplemental Figure S4. There was a statistically significant difference in the tumor mutational burden (TMB) results between dMMR and pMMR groups, excluding the case with POLE mutation (P = 0.003 by Mann-Whitney U test). [file 12885_2023_11607_MOESM1_ESM.docx]

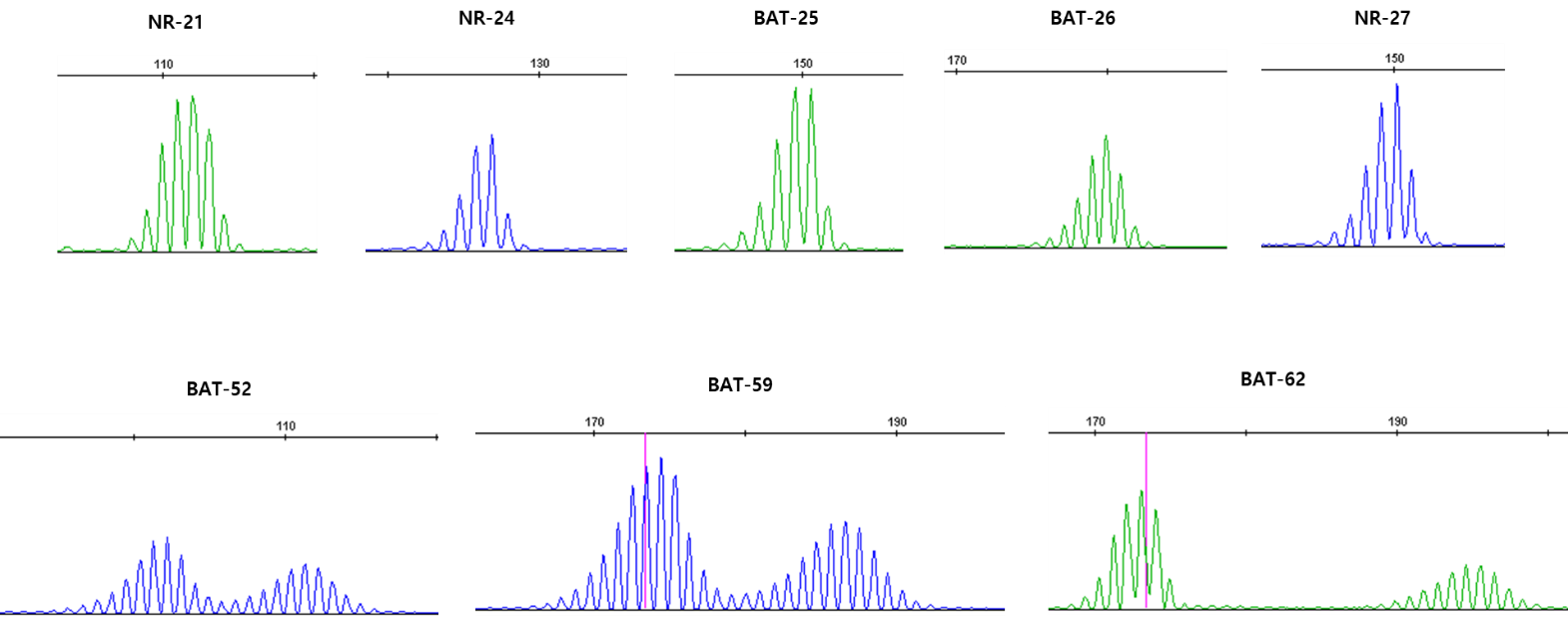


Supplemental Figure S1. Representative electropherogram of a normal tissue showing heterozygosity in all long mononucleotide repeat markers.


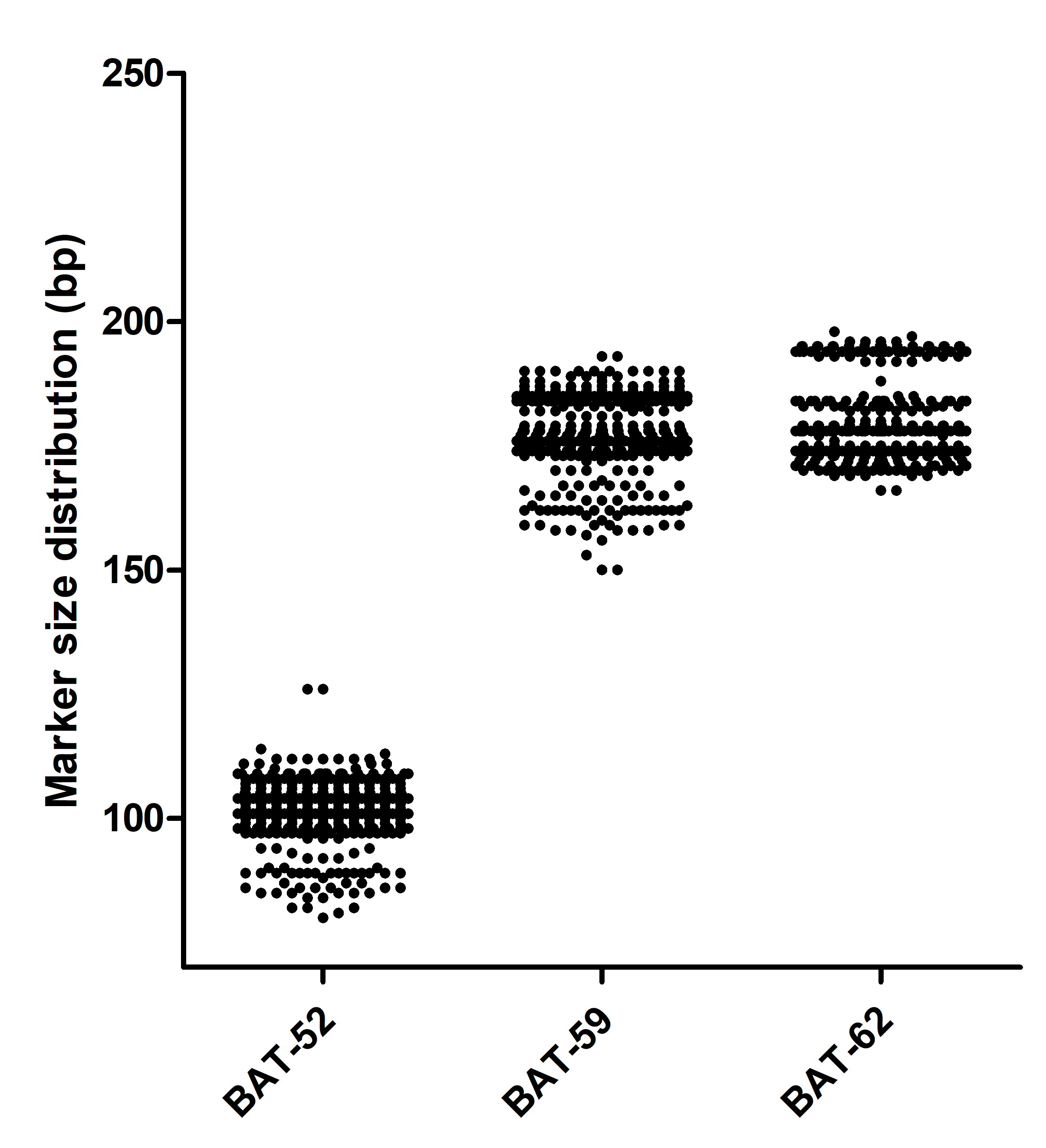


Supplemental Figure S2. The distribution of marker size in each long mononucleotide repeat (LMR) marker (BAT-52, BAT-59, and BAT-62) using 300 cases of normal tissues.


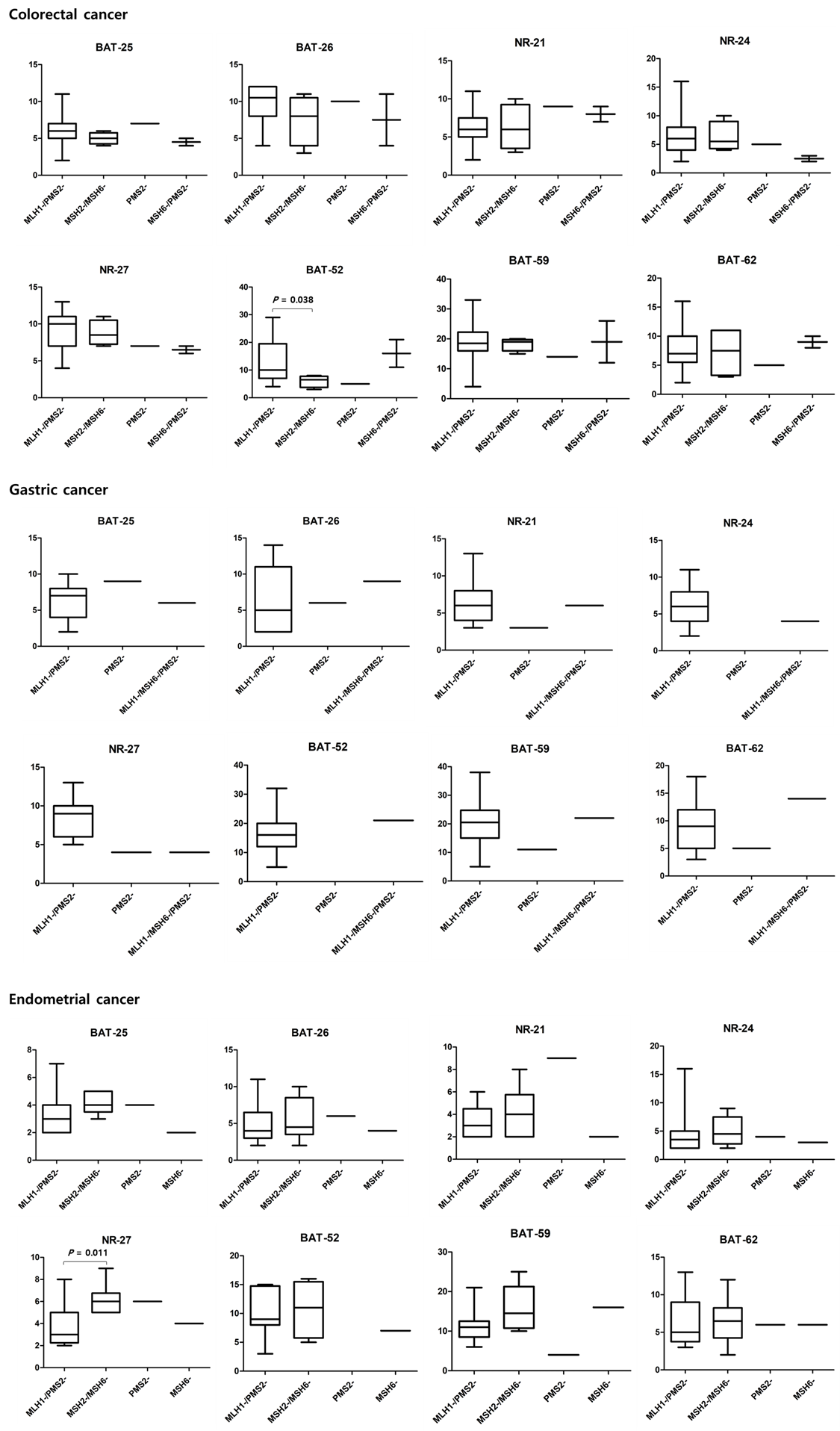


Supplemental Figure S3. Box and whiskers plot showing the median, max, min and 1st and 3rd quartile of the size of allelic changes for MSI detection in individual marker according to the patterns of deficient mismatch repair proteins. There was a statistically significant difference between MLH1-/PMS2- and MSH2-/MSH6- for BAT-52 in colorectal cancer (*P* = 0.038) and for NR-27 in endometrial cancer (*P* = 0.011), using the Kruskal–Wallis test.


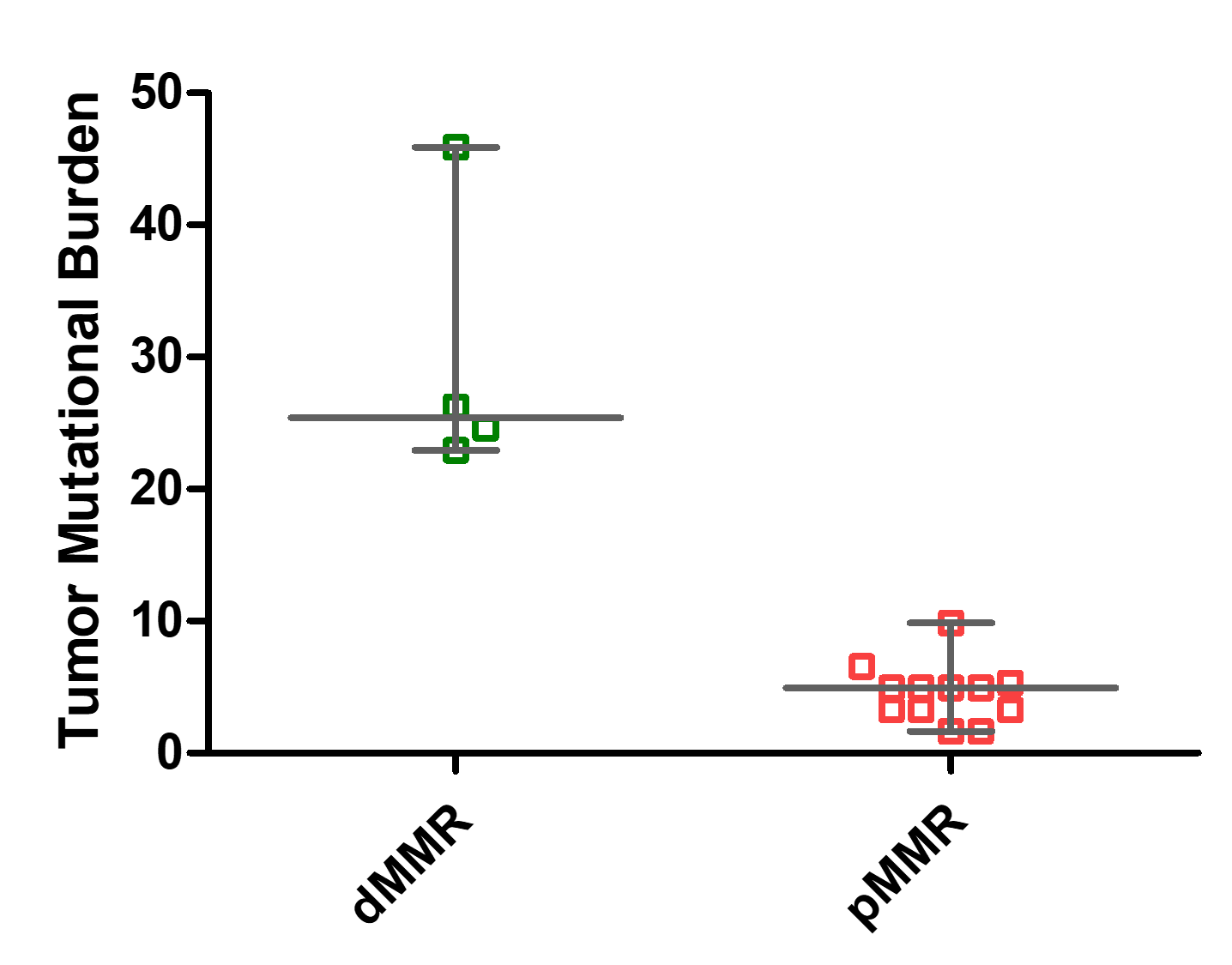


Supplemental Figure S4. There was a statistically significant difference in the tumor mutational burden (TMB) results between dMMR and pMMR groups, excluding the case with POLE mutation (*P* = 0.003 by Mann-Whitney U test).
